# Human practices promote presence and abundance of disease-transmitting mosquito species

**DOI:** 10.1038/s41598-020-69858-3

**Published:** 2020-08-11

**Authors:** Maarten Schrama, Ellard R. Hunting, Brianna R. Beechler, Milehna M. Guarido, Danny Govender, Wiebe Nijland, Maarten van ‘t Zelfde, Marietjie Venter, Peter M. van Bodegom, Erin E. Gorsich

**Affiliations:** 1 Institute of Environmental Sciences, Leiden University, Leiden, The Netherlands; 2 School of Biological Sciences, University of Bristol, Bristol, UK; 3 Department of Biomedical Sciences, College of Veterinary Medicine, Oregon State University, Corvallis, OR USA; 4 Department of Medical Virology, University of Pretoria, Pretoria, South Africa; 5 Scientific Services, SANPARKS, Kruger National Park, Skukuza, South Africa; 6 Department of Physical Geography, University of Utrecht, Utrecht, The Netherlands; 7 School of Life Sciences, University of Warwick, Coventry, UK; 8 The Zeeman Institute for Systems Biology and Infectious Disease Epidemiology Research, University of Warwick, Coventry, UK

**Keywords:** Freshwater ecology, Community ecology, Ecological epidemiology, Ecosystem ecology, Freshwater ecology, Infectious diseases, Environmental impact

## Abstract

Humans alter the environment at unprecedented rates through habitat destruction, nutrient pollution and the application of agrochemicals. This has recently been proposed to act as a potentially significant driver of pathogen-carrying mosquito species (disease vectors) that pose a health risk to humans and livestock. Here, we use a unique set of locations along a large geographical gradient to show that landscapes disturbed by a variety of anthropogenic stressors are consistently associated with vector-dominated mosquito communities for a wide range of human and livestock infections. This strongly suggests that human alterations to the environment promote the presence and abundance of disease vectors across large spatial extents. As such, it warrants further studies aimed at unravelling mechanisms underlying vector prevalence in mosquito communities, and opens up new opportunities for preventative action and predictive modelling of vector borne disease risks in relation to degradation of natural ecosystems.

## Introduction

Habitat destruction, chemical pollution, and climate change are ongoing human disturbances^1^ that have resulted in world-wide shifts in insect communities^2^. While most insects are declining^2^, many mosquito species are thriving^3,4^. Recent laboratory and mesocosm studies provided important clues suggesting that anthropogenic disturbances can promote population growth of several pathogen-transmitting mosquito species, or disease vectors^5-8^, and potentially mediate interactions between mosquito species^9^, their hosts^10^, their pathogens^11,12^, and their predators^13^. This hints that, if these patterns hold true for natural systems, the way humans influence their local environment presents a critical driver of disease risk^14^.

Field data in natural systems has largely focused on the influence of climate—temperature and precipitation—in driving the abundance of single species or vector groups^15-19^. These studies constitute an important foundation for mechanistic models and risk maps to anticipate disease outbreaks such as malaria, chikungunya and Zika^20,21^. Mosquito abundance and composition can also vary across locations and land use types^22-27^. However, many comparisons rely on opportunistic sampling across different time periods or targeted sampling at locations to maximize collections^28^, but not always^29,30^. Here, we use a paired sampling design to show that human activities beyond climate are strongly associated with high abundances of known vectors across large spatial extents.

**Figure 1:**
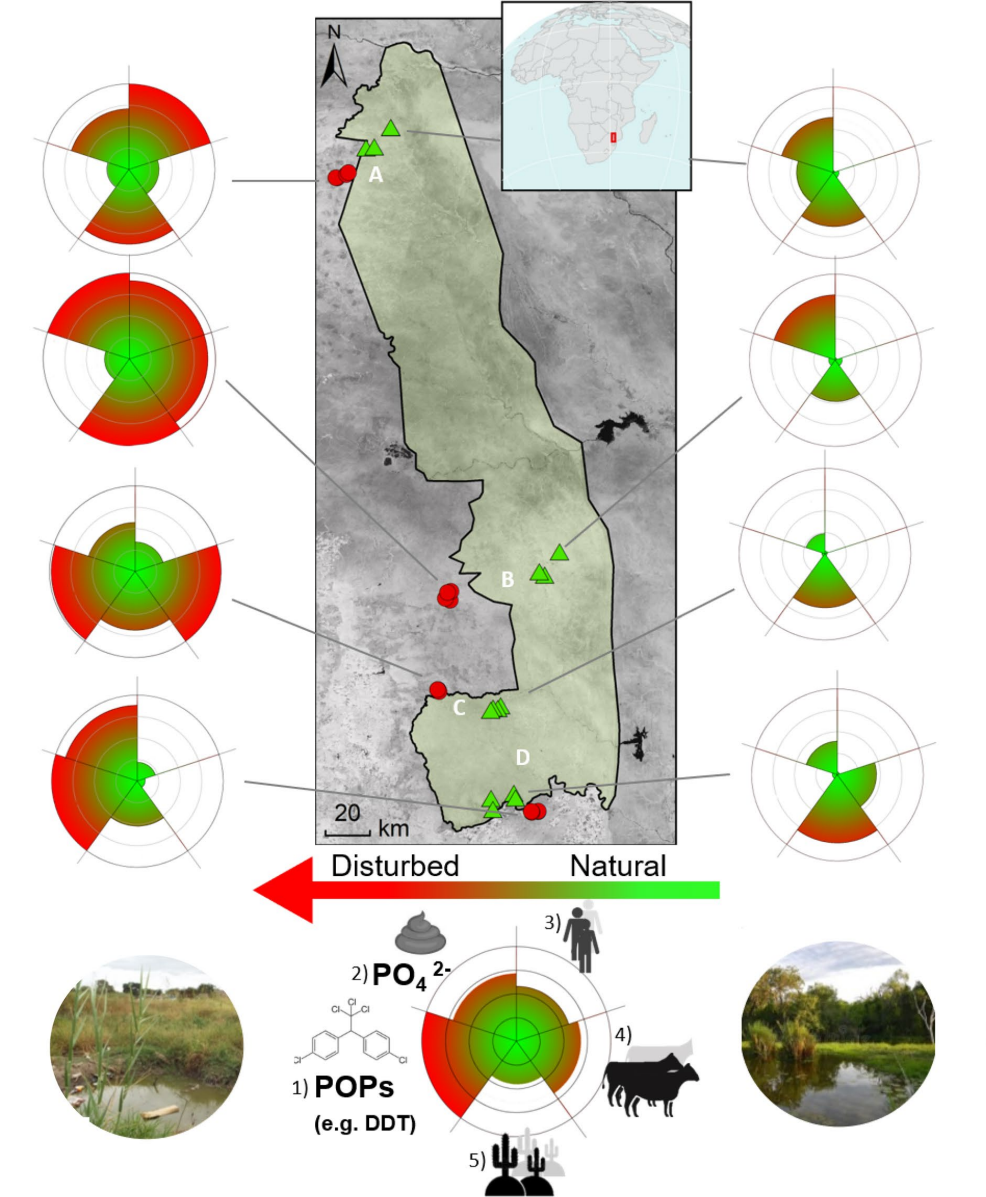
Variation in anthropogenic pressures comparing waterbodies inside (green triangles) and outside KNP (red symbols). Each radar plot represents five pressures; intensity runs from low (green) to high (red) and was rescaled by dividing by the maximum: (1) pesticide concentrations (POPs); (2) aquatic eutrophication; (3) human population density; (4) ungulate biomass; (5) percentage bare or sparsely vegetated area. A–D refer to the different regions: Punda Maria (**A**), Satara (**B**), Skukuza (**C**) and Malelane (**D**). Maps were constructed in ArcGIS 10.5. Photos: Maarten Schrama.

Kruger National Park (KNP) is the largest natural reserve in South Africa and is fringed with rural and urbanizing areas. The mosaic of waterbodies along its 400 km north–south gradient provides a unique opportunity to assess the effect of human disturbances on mosquito population dynamics and community composition. We simultaneously sampled representative waterbodies within paired sites inside and outside the national park in four regions (Fig. 1); the paired sites have similar geomorphology and climatic conditions (Table S1, Figure S1) but differ in how they are affected by humans. To obtain an overall assessment of anthropogenic disturbance, we quantified 5 ubiquitous anthropogenic pressures^1^: (1) organophosphate pesticide abundance, (2) eutrophication, (3) human population density, (4) ungulate biomass and (5) vegetation loss, at each of the paired locations, all of which have separately been shown to influence mosquito populations^5,6,10,24^.

## Results and discussion

The five pressures were consistently higher outside vs. inside KNP: (1) mean concentration of organophosphates was 196.1 outside vs. 3.5 µg kg^−1^ inside (F_(1,15)_ = 9.12; *p* < 0.01); (2) eutrophication measured as the mean dissolved phosphate concentration was 0.91 outside vs, 0.60 mg P L^−1^ inside *(*F_(1,31)_ = 4.6*; p* = 0.04); (3) human population density was 844 outside vs. 0.06 indiv. km^−2^ inside (F_(1,6)_ = 307.3; *p* < 0.01); (4) biomass of ungulate livestock was 1,186 outside vs. 321 kg km^−2^ inside (F_(1,31)_ = 10.7; *p* < 0.01); (5) the percentage cover of bare and sparsely vegetated soil was 62% outside vs. 42% inside KNP (F_(1,31)_ = 6.38; *p* = 0.02). The magnitude of these differences varied between regions (Table S2). These pressures generate a composite measure of disturbance (Fig. 1) and confirm that water bodies outside KNP are consistently more impacted by anthropogenic pressures.

**Figure 2:**
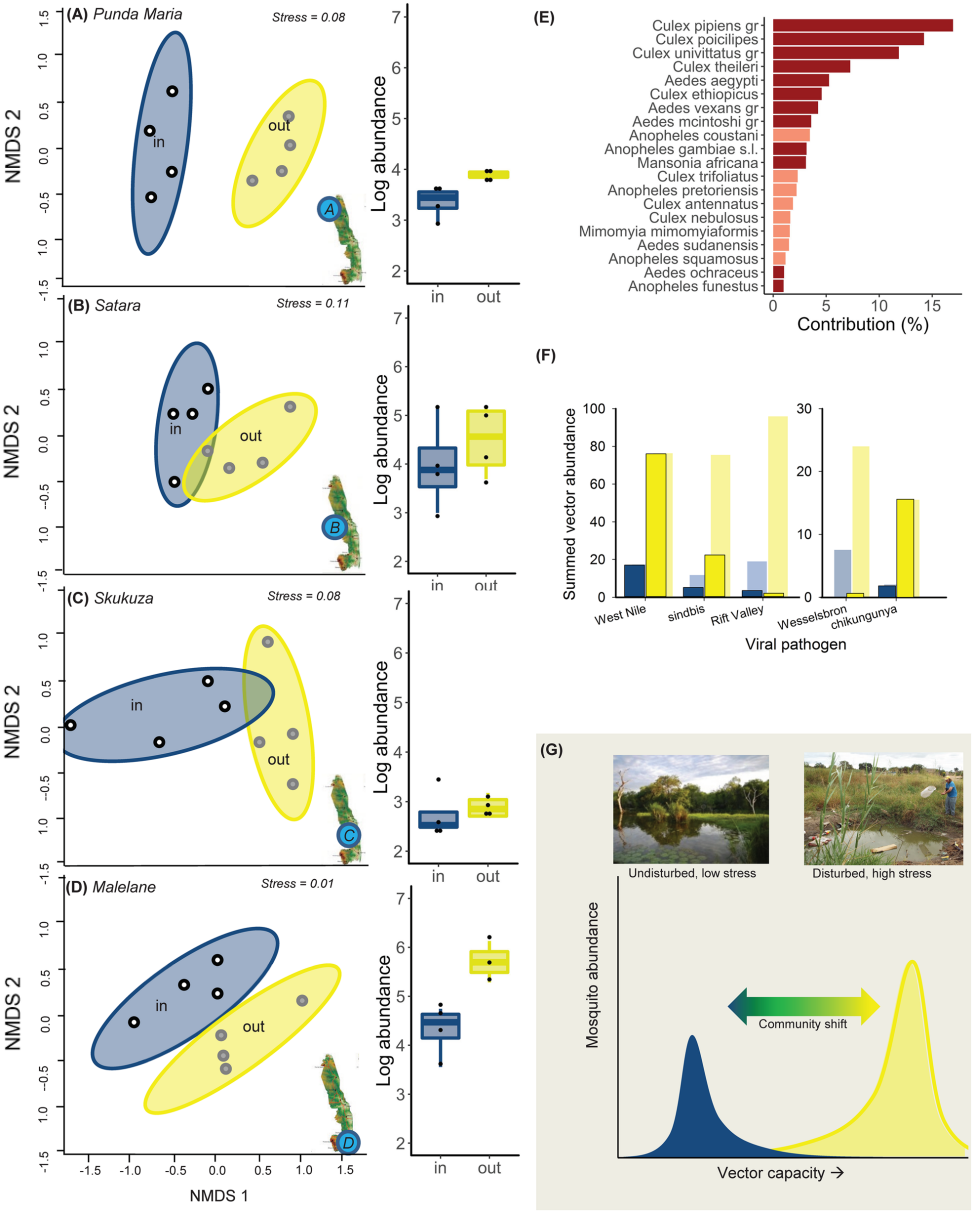
Effects of human-induced land use change on mosquito composition and abundance*.* (**A**–**D**): *NMDS and box plots showing shifts in composition and abundance between sites inside (“in”) and outside KNP (“out”).* Points further apart are more dissimilar; circles depict 95% confidence intervals; stress values represent goodness of fit. (**E**) *SIMPER showing the relative species contribution to changes in species composition between sites inside and outside KNP.* Only species that explain > 1% of the variation are depicted. Dark red bars: known disease vectors; light red bars: non-vectors^32^. (**F**) *Summed abundance of primary (outlined bars with saturated colors) and secondary vectors*^32^ (*bars with light shades and no outline*) (**G**) *Conceptual diagram synthesizing how anthropogenic stressors may drive vector capacity by shifting mosquito species communities* from low-stressor (blue) towards high-stressor conditions (yellow).

To quantify whether these higher levels of disturbance are linked to changes in mosquito communities, we trapped 3,918 females of 39 mosquito species and species complexes (Diptera: Culicidae). The paired trapping design (Fig. 1) allowed us to simultaneously collect mosquitoes from waterbodies inside and outside KNP^31^. Mosquito abundance outside the national park was on average 2.9 times higher (range: 1.5–10 times higher) than inside KNP (F_(1,24)_ = 17.3, *p* < 0.001; Fig. 2A–D). This pattern resembles mosquito abundances along gradients of human disturbance in the Mediterranean^24^ and tropical rainforests in Thailand^29^. More importantly, we observed pronounced shifts in the species composition of mosquitoes inside vs. outside KNP (ANOSIM: r = 0.15; *p* < 0.001) for each of the four geographical regions (Fig. 2A–D, Table S3). Despite differences in overall richness (35 species outside vs 30 inside KNP) and abovementioned differences in species composition, we observed no differences between different alpha diversity metrics inside vs. outside KNP (species richness, Shannon’s J, Shannon’s H’max and Simpson’s evenness; Figures S2A–C). Known vectors of human and livestock diseases were more abundant outside vs. inside KNP; these species explained 79% (± SD 3) of the variation in communities between paired regions (Fig. 2E; SIMPER: r = 0.48; *p* < 0.001). This pattern was consistent for each of the regions (Malelane 82%, Satara 78%, Punda Maria 76%, Skukuza 77%; Figures S3A–D, S4). It was also largely consistent across vectors and infections: *Aedes aegypti,* a vector for dengue, chikungunya, yellow fever and Zika^32^, was more common outside KNP. Multiple *Culex* species were also more common outside, including vectors for West Nile, sindbis, Wesselsbron and filariasis (Fig. 2E–F). Only two vector species, *Ae. vexans* and *Cx. theileri,* vectors from Rift Valley fever^32^, were more abundant inside KNP. These patterns are in line with studies carried out along gradients of deforestation, which have been shown to lead to increased presence of malaria vectors such as *An. gambiae* and *An. darlingi*^18,33-35^.

Together, these results show that human disturbances are strongly associated with increased mosquito abundances and shifts in community composition towards known disease vectors, as illustrated in conceptual Fig. 2G. They highlight the experimentally-demonstrated importance of changes in environmental conditions^5,6^ by identifying its consistent pattern across large geographic extents and climatic conditions. These results emphasize that anthropogenic disturbances to the landscape and the larval environment represent an important mechanism driving vector distributions. Given the global extent and intensity of the investigated anthropogenic pressures^1^, these results are likely relevant for a wide array of vector-borne pathogens and provides a mechanism for the association between ecosystem degradation and disease^36,37^. This raises important questions on how different human activities drive vector prevalence in mosquito communities, and presents new opportunities for targeted preventative action as well as predictive modelling of vector borne disease risks in relation to ecosystem services.

## Methods

Mosquito trapping and identification have been described previously^31^; mosquito sampling occurred across 4 regions and 112 trapping nights, with simultaneous collections for 3–4 consecutive nights at all sampling points in a region (4 inside and 4 outside KNP). Sampling points within a region were selected based on multiple criteria. The primary selection criterion was to sample from waterbodies that were representative of the region, including a diversity of wetlands rather than those with the highest catch rates^28^. Additional criteria stipulated that the water bodies were at least 1 km away from one another to avoid sampling mosquitoes from adjacent water bodies, as mean mosquito dispersal distances range from 35 m to 1.4 km^31^. In this setup, we follow the ecosystem boundaries framework^38^ and quantified 5 pressures associated with human impacts in the different ecosystems. The concentration of different persistent organophosphates (POPs, e.g., DDT and breakdown products) were measured at 2 sampling points per region using multi-residue analysis (GC-ECD and GC–MS) by the African Research Council (Roodeplaat, South Africa). We determined phosphate levels (PO_4_^3−^) using a photospectrometer (Merck Spectroquant Nova 60) in one litre of water, which was composed of 20 subsamples of 50 ml per sampling point. The fraction of bare and sparsely vegetated area at each sampling point was assessed using satellite data derived from the Sentinel-2 sensor acquired in January 2017. The 2-band Enhanced Vegetation Index (EVI)^39^ was calculated from a monthly maximum EVI composite avoiding atmospheric disturbances. To derive the percentage of barren and sparsely vegetated areas EVI thresholds of 0.15 and 0.35 were used respectively. The proportion of barren and sparsely vegetated pixels (10*10 m) within a 150 m radius around each sample point was taken as a representative sample of the vegetation cover for each of the trapping locations. The fraction of bare and sparsely vegetated area was assessed using monthly maximum Enhanced Vegetation Index (EVI; using Sentinel-2data Jan–March 2017). In a radius of 150 m around each sample point, we estimated the percentage of pixels (10*10 m) between EVI thresholds of 0.15 and 0.35, respectively. Animal densities at each sampling point were determined using the gridded livestock of the world map (fao.org/livestock-systems/global-distributions; resolution: 0.05′*0.05′ degrees ~ 5*5 km) and the 2018 KNP African buffalo *(Syncerus caffer)* counts. All numbers were transferred to a raster file with 0.05′*0.05′ grid cells, after which the average density was calculated in a buffer ring of 2 km diameter around each sampling point. Human population densities for each of the regions inside and outside KNP were determined using the 2011 population census using data at the scale of the municipality (https://www.statssa.gov.za).

To test for differences in mosquito abundance inside vs. outside KNP, we used general linear models comparing the number of mosquitos collected at each site aggregated across each night. Region (Punda Maria, Satara, Skukuza and Malelane), disturbance (inside vs. outside KNP) and their interaction were included as main effects. The number of mosquitoes collected was overdispersed, log-transformed for normality, and assessed using Quantile Quantile-plots and a Levene's test (*P* = 0.06). Independence assumptions of the regression model were evaluated using plots of model residuals by location and distance between sites.

Differences in community composition inside vs. outside KNP were tested based on the species composition data aggregated across all nights at each site, because multiple trapping nights are needed to capture rare species^26,31^. The data were analysed using a non-parametric analysis of similarities analysis (ANOSIM) and visualized with non-metric multidimensional scaling (NMDS). The ANOSIM analysis is a non-parametric test for differences in mosquito communities among traps that compares the ranks of Bray–Curtis dissimilarity measures from samples collected inside vs. outside KNP^40,41^. A SIMPER analysis was used to assess which taxa are responsible for shifts in community composition^42^. Overall patterns of richness and diversity (Shannon’s J, Shannon’s H’max and Simpson’s evenness) are also reported (Fig. S2A–C), but the SIMPER analysis provides is more appropriate, because it was developed to identify the species responsible for shifts in community measures^42^. All analyses were conducted in R using the lme4 and vegan packages^43,44^.

## Supplementary information

Supplementary information
