# Supplementary material for: Human practices promote presence and abundance of disease-transmitting mosquito species
Source: Sci Rep. 2020 Aug 11;10:13543. doi: 10.1038/s41598-020-69858-3 (PMC7421943; doi:10.1038/s41598-020-69858-3)
Supplement: Supplementary file 1 — Supplementary information [file 41598_2020_69858_MOESM1_ESM.docx]

**Supplementary material**: *“Human practices promote presence and abundance of disease-transmitting mosquito species”*

Maarten Schrama^1^*, Ellard R. Hunting ^2^, Brianna R. Beechler^3^, Milehna M. Guarido^4^, Danny Govender^4,5^, Wiebe Nijland^6^, Maarten van ‘t Zelfde^1^, Marietjie Venter^4^, Peter M. van Bodegom^1^, Erin E. Gorsich^1,7^

*^1^Institute of Environmental Sciences, Leiden University, Leiden, The Netherlands*

*^2^School of Biological Sciences, University of Bristol, Bristol, UK*

*^3^Department of Biomedical Sciences, College of Veterinary Medicine, Oregon State University, Corvallis, Oregon, USA.*

*^4^ Department of Medical Virology, University of Pretoria, Pretoria, South Africa*

*^S^SANPARKS, Scientific Services, Kruger National Park, South Africa*

*^6^Department of Physical Geography, University of Utrecht, The Netherlands*

*^7^School of Life Sciences and The Zeeman Institute for Systems Biology & Infectious Disease Epidemiology Research, University of Warwick, Coventry, UK.*

***Table S1.*** *Climate variables: Mean annual temperature (Mean Temp.) Mean maximum annual temperature (Max Temp) and precipitation (Rainfall) at each of the study locations.* Locations are ranked north-south; sites towards the north were dryer and warmer. There was no significant inside vs. outside difference for these climatic variables but the study locations differed from each other in each of the variables.

| **Site Name** | **Inside/**  **outside** | **Mean Temp.**  **(⁰C)** | **SD** | **Max Temp**  **(⁰C)** | **SD** | **Rainfall**  **(mm)** | **SD** |
| --- | --- | --- | --- | --- | --- | --- | --- |
| *Punda Maria* | *Inside* | 23.03 | 0.39 | 30.19 | 0.41 | 556.50 | 14.15 |
|  | *Outside* | 22.94 | 0.03 | 30.36 | 0.16 | 578.25 | 9.54 |
|  |  |  |  |  |  |  |  |
| *Satara* | *Inside* | 22.23 | 0.14 | 29.56 | 0.34 | 574.75 | 33.56 |
|  | *Outside* | 21.66 | 0.03 | 28.47 | 0.07 | 553.75 | 21.69 |
|  |  |  |  |  |  |  |  |
| *Skukuza* | *Inside* | 21.67 | 0.03 | 29.06 | 0.18 | 613.00 | 3.65 |
|  | *Outside* | 21.74 | 0.03 | 28.73 | 0.06 | 656.50 | 3.79 |
|  |  |  |  |  |  |  |  |
| *Malelane* | *Inside* | 21.91 | 0.10 | 28.80 | 0.21 | 627.25 | 24.06 |
|  | *Outside* | 22.28 | 0.04 | 29.31 | 0.09 | 636.50 | 11.00 |
|  |  |  |  |  |  |  |  |
|  |  |  |  |  |  |  |  |

***Table S2.*** *Averages and ranges for each of the five stressors in Figure 1 of the main text.* Locations are ranked north-south.

| **Site Name** | **Inside/**  **outside** | **Fraction barely vegetated soil**  **Average (range)** | **Herbivore density (kg/km^2^; range)** | **Human population**  **Density (ind/km^2^)** | **Pesticide concentration log(ug/kg) (range)** | **PO4 concentration (mg/l) (range)** |
| --- | --- | --- | --- | --- | --- | --- |
| *Punda Maria* | Inside | 0.52 (0.37-0.59) | 128 (61.8-320) | 0.07 | 1.15 (0.27-1.42) | 0.78 (0.13-1.54) |
|  | Outside | 0.70 (0.35-0.77) | 714 (0-1523) | 1366 | 0.66 (0.36-0.88) | 0.82 (0.07-2.00) |
|  |  |  |  |  |  |  |
| *Satara* | Inside | 0.39 (0.13-0.91) | 149 (103-219) | 0.07 | 0.18 (0.17-0.19) | 0.88 (0.10-1.31) |
|  | Outside | 0.80 (0.75-0.86) | 1841 (878-2012) | 1242 | 0.79 (0.72-0.86) | 1.17 (0.26-3.05) |
|  |  |  |  |  |  |  |
| *Skukuza* | Inside | 0.51 (0.43-0.57) | 76.0 (32.0-143) | 0.07 | 0.07 (0.00 – 0.17) | 0.66 (0.09-1.51 |
|  | Outside | 0.55 (0.50-0.62) | 2006 (1770-2112) | 467 | 2.57 (2.47-2.64) | 0.27 (0.05-0.54) |
|  |  |  |  |  |  |  |
| *Malelane* | Inside | 0.42 (0.13-0.88) | 932 (64.9-2016) | 0.07 | 0.11 (0.02-0.18) | 0.45 (0.09-1.38) |
|  | Outside | 0.64 (0.52-0.99) | 184 (171-196) | 308 | 2.61 (1.09-2.90) | 1.03 (0.25-2.33) |
|  |  |  |  |  |  |  |
|  |  |  |  |  |  |  |

***Table S3.*** *Aggregated number of females for each of the mosquito species collected at the four locations inside (park locations) and the neighbouring locations outside the park (rural locations).*

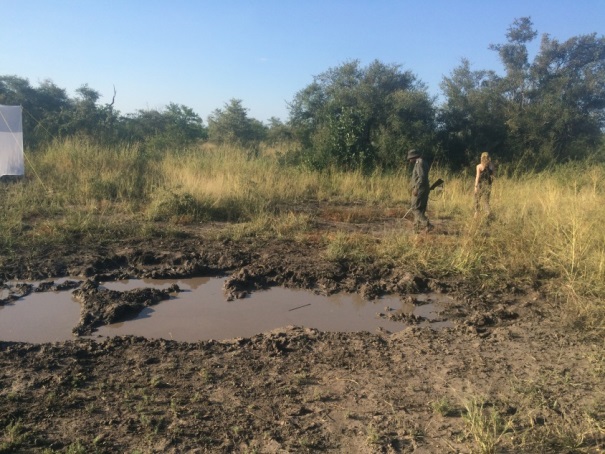

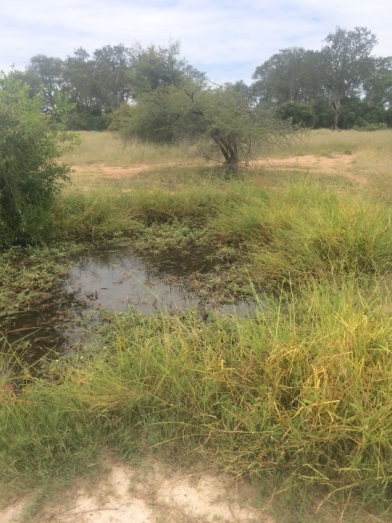


Skukuza


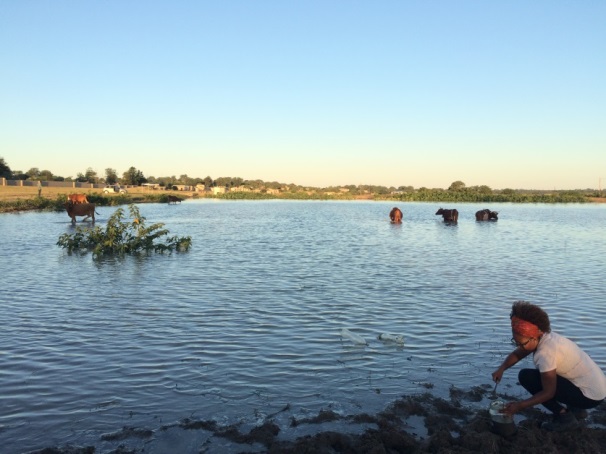


Welverdiend


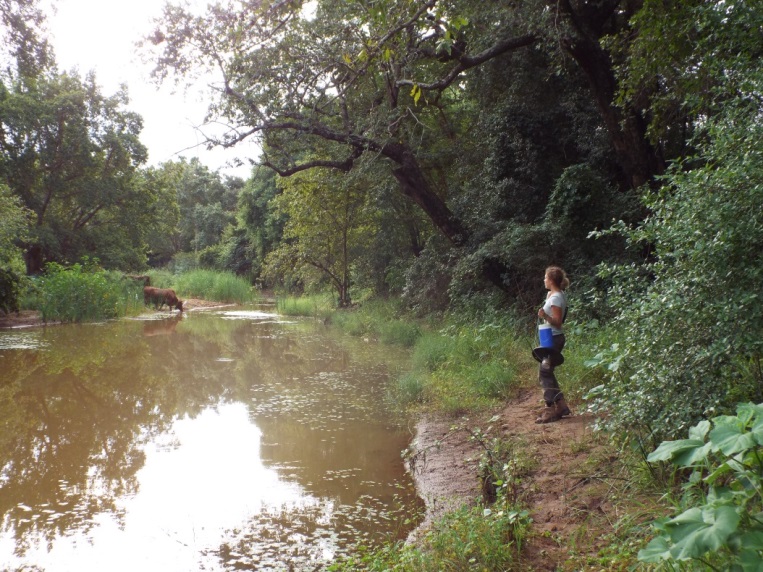


Malamulele


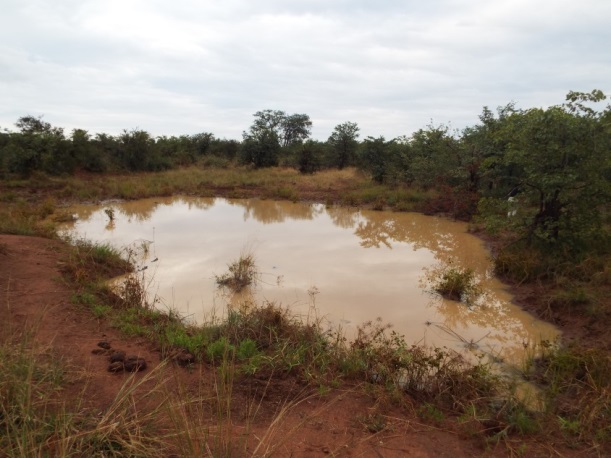


Punda Maria


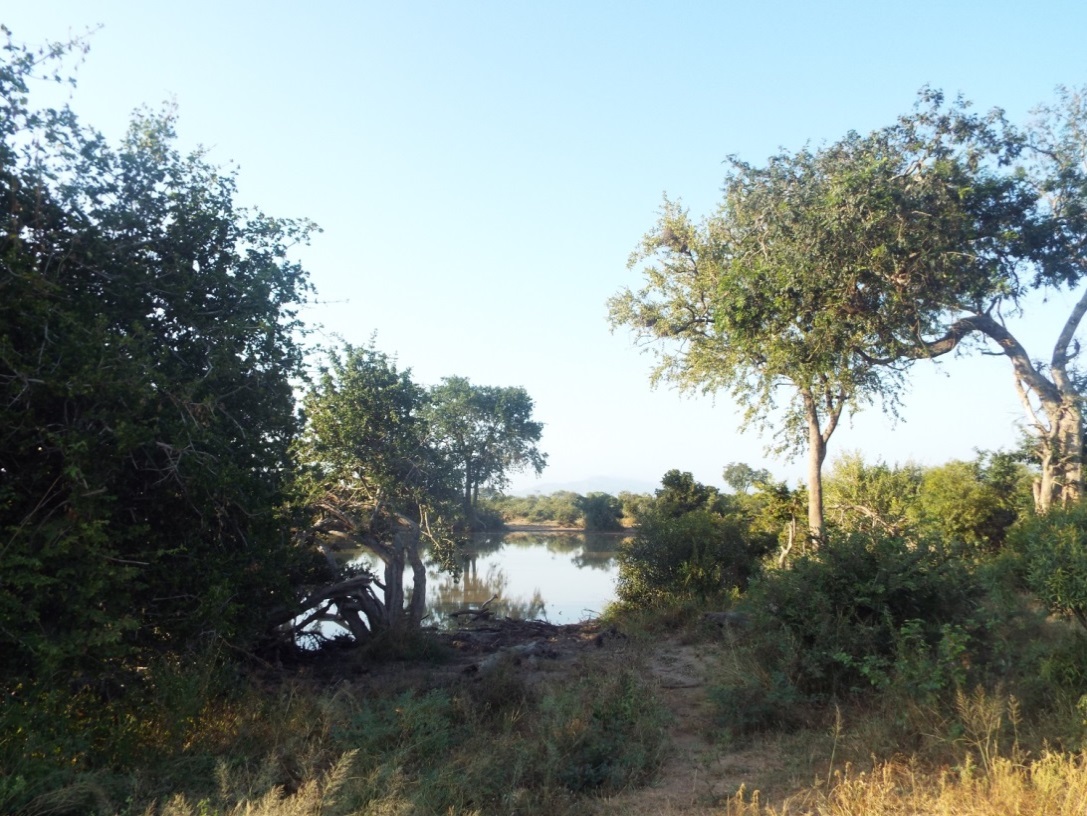


Malelane


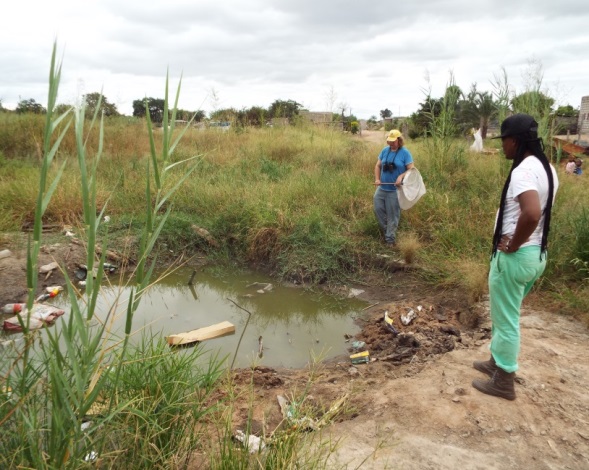


Hectorspruit

Satara


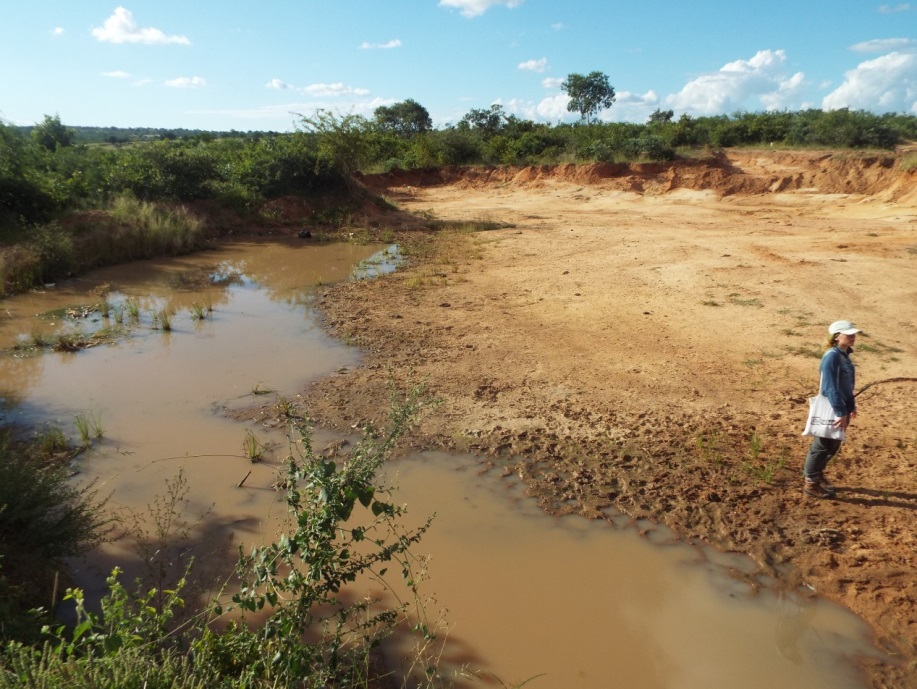


Cork

Inside the Kruger Park

Outside the Kruger Park

**Figure S1.** *Pictures of a subset of the study locations in each of the 4 paired regions inside (top row) and outside (bottom row) Kruger National Park*. See Fig 1 for geographical location of sites. All pictures taken by M. Schrama.


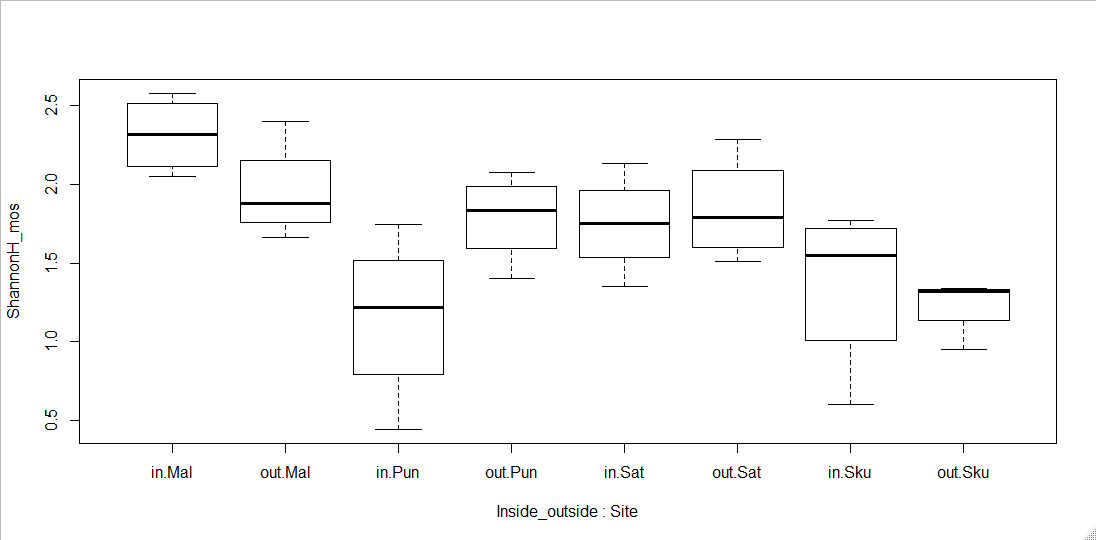

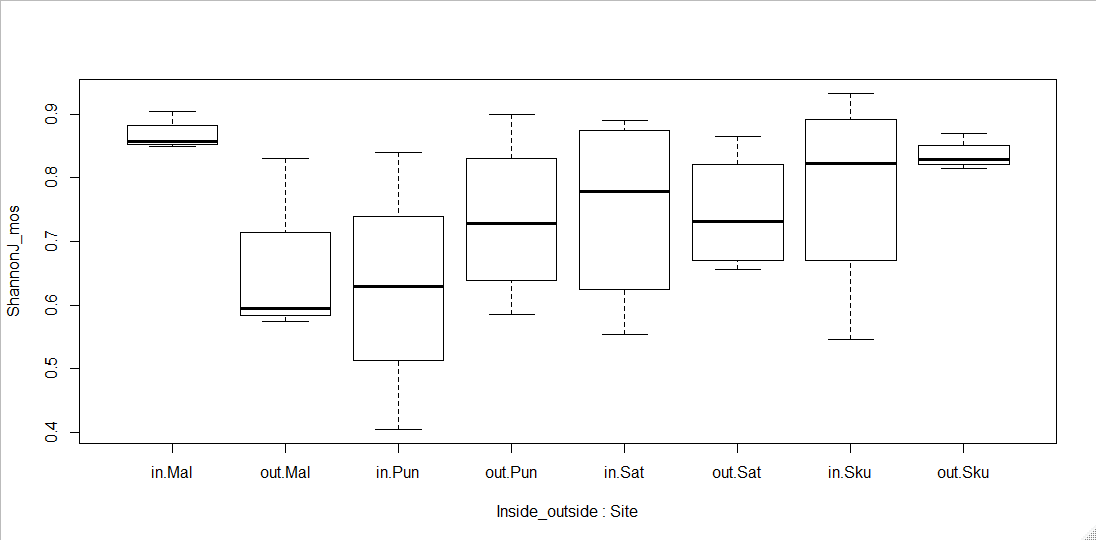

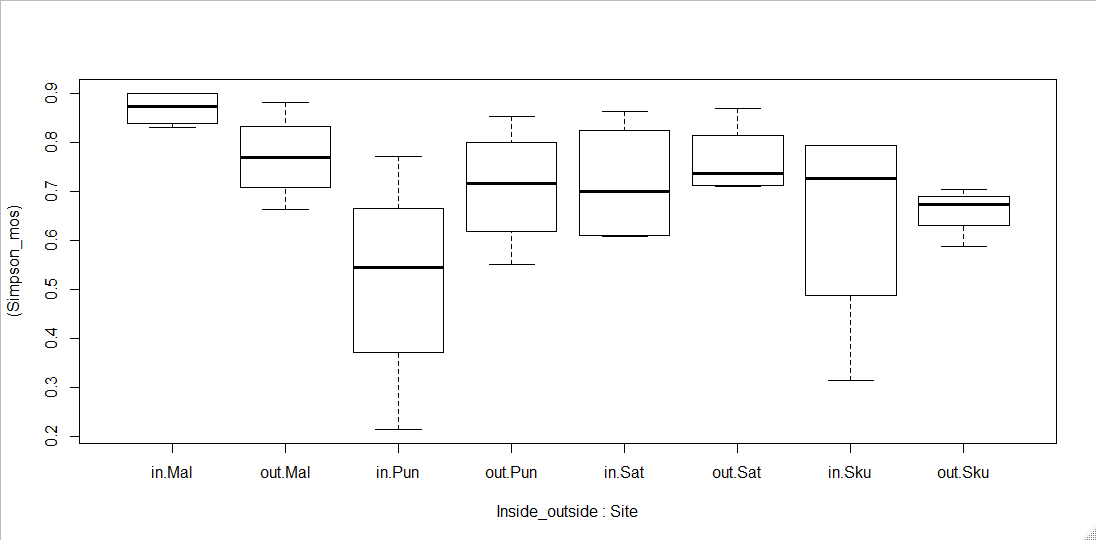


**Figure S2.** *Boxplots of species diversity estimates between the different locations inside vs. outside the park.* A) Shannon’s J B) Shannon’s H’max and C) Simpson’s evenness index. Significance for each factor is indicated on the left of each panel.
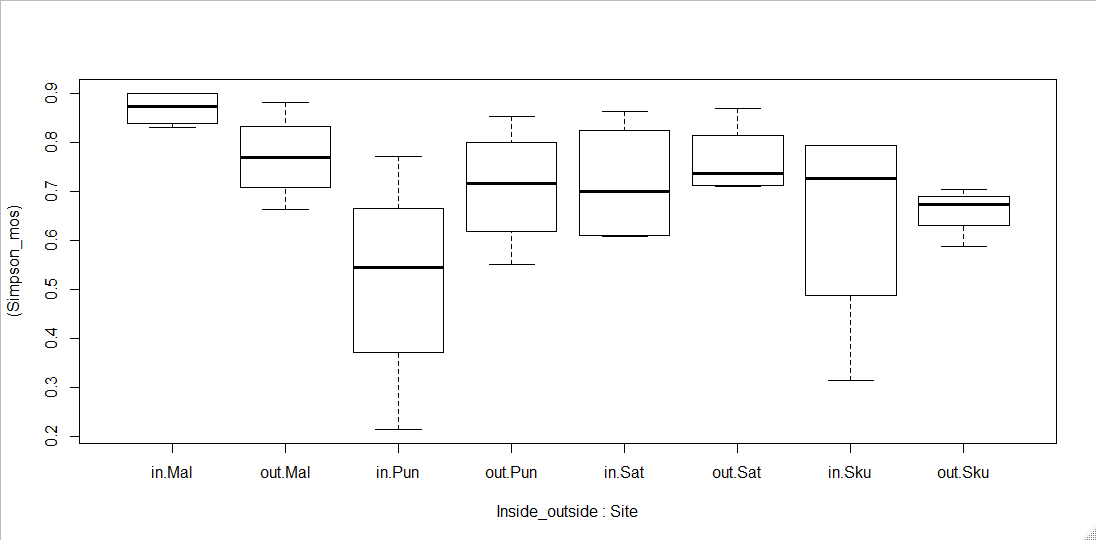


Inside_outside F_(1,24)_ 1.0011 P = 0.33

Site F_(3,24)_ 4.4816 P = ***0.01*** *

Ins_Out*Site F_(3,24)_ 1.4620 P = 0.25

Inside_outside F_(1,24)_  6.0460 P = 0.06

Site F_(3,24)_ 2.5258 ***P = 0.02****

Ins_Out*Site F_(3,24)_ 2.6087 P = 0.08`

Inside_outside F_(1,24)_ 1.9519 P= 0.18

Site F_(3,24)_ 7.7841 ***P < 0.001 ****

Ins_Out*Site F_(3,24)_ 2.7097 P = 0.07


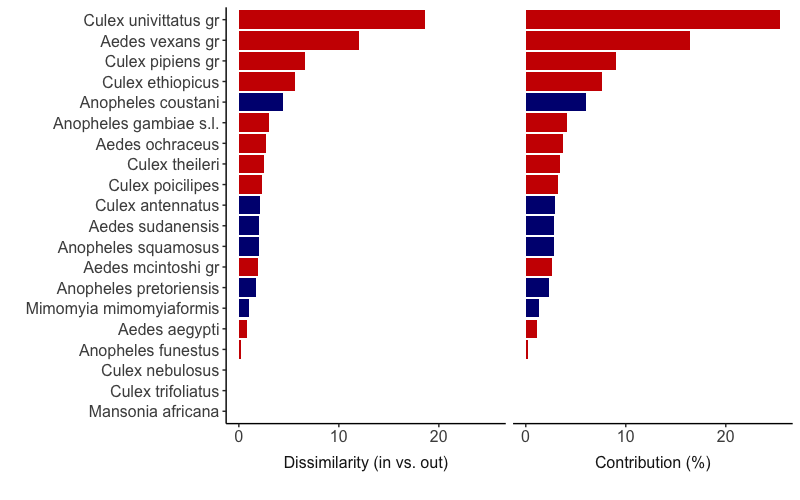

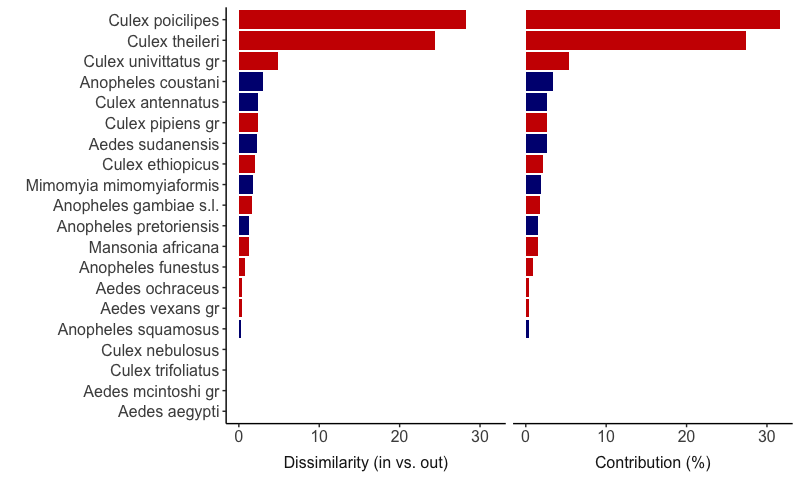


B.

A.


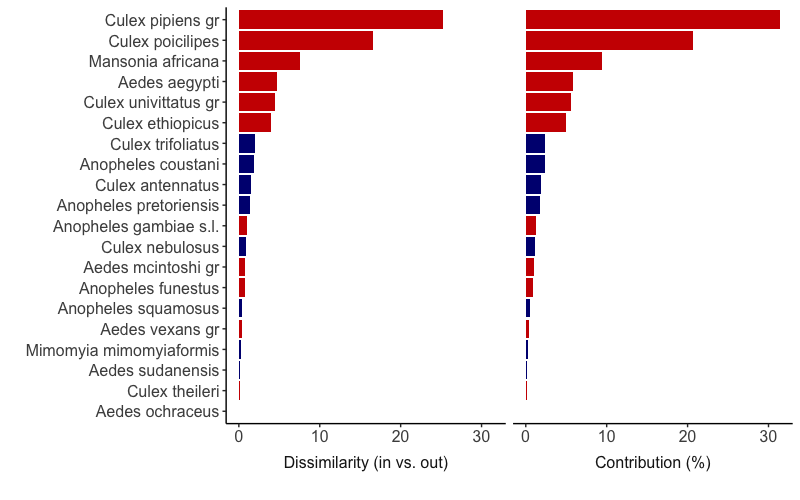

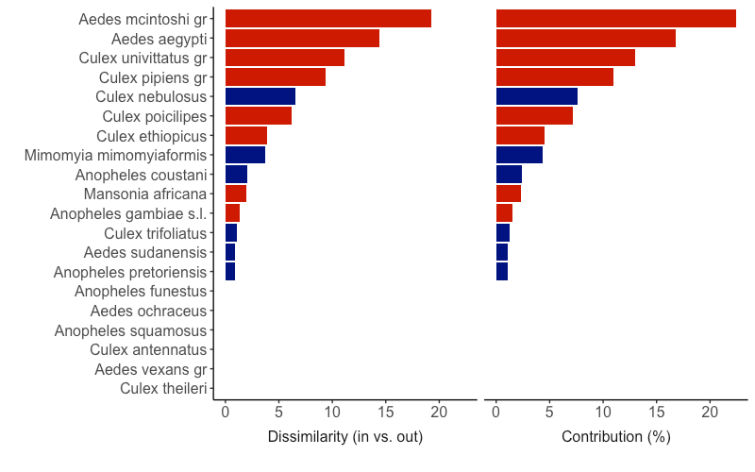


D.

C.

***Figure* S3** *Relative dissimilarity and contribution to observed differences in species composition in each of the four paired trapping locations* A) Punda Maria, B) Satara, C) Skukuza and D) Malalane as identified using a SIMPER analysis. Only species that contribute > 1% are shown. Blue bars show non-vector species, red bars indicate disease vectors for the commonly mentioned disease vectors in southern Africa for diseases such as rift valley fever, sindbis, West Nile, dengue, chikungunya and wesselsbron within the Anophelinae and the Culicinae^1^.


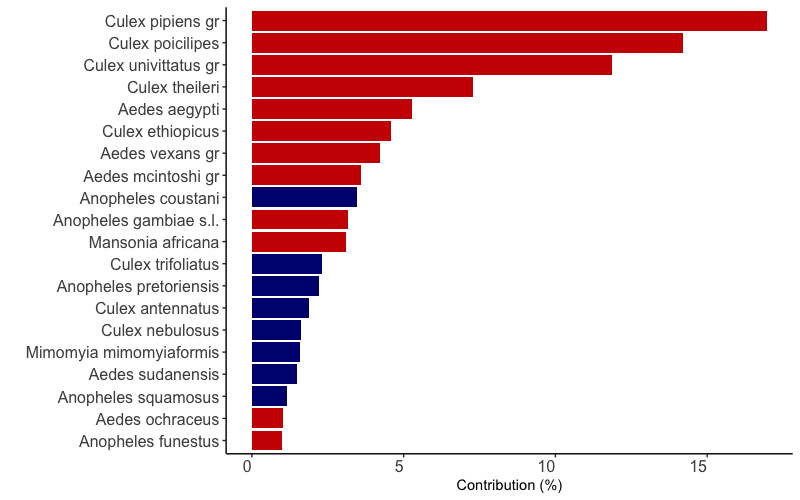
 **Figure S4.** *Overall relative contribution of the different species to differences in mosquito species composition inside and outside the park. Legend and analysis similar to Figure S2.*
